# Supplementary material for: Monobody adapter for functional antibody display on nanoparticles for adaptable targeted delivery applications
Source: Nat Commun. 2022 Oct 11;13:5998. doi: 10.1038/s41467-022-33490-8 (PMC9553936; doi:10.1038/s41467-022-33490-8)
Supplement: Supplementary file 1 — Supplementary Information [file 41467_2022_33490_MOESM1_ESM.pdf]

Supplementary Information

Title: Monobody adapter for functional antibody display on nanoparticles for adaptable targeted delivery applications

Authors: C. Albert[1, Ŧ], L. Bracaglia[1, Ŧ], A. Koide[2,3], J. DiRito[4], T. Lysyy[4], L. Harkins[1], C. Edwards[4], O. Richfield [1,4], J. Grundler[1], K. Zhou[5], E. Denbaum[2], G. Ketavarapu[2], T. Hattori[2], S. Perincheri[6], J. Langford[4], A. Feizi[1], D. Haakinson[4], S.A. Hosgood[7], M.L. Nicholson[7], J.S. Pober[8], W.M. Saltzman[1], S. Koide[2,9,*], G. T. Tietjen[1,4,*]

[Ŧ] These authors contributed equally; [1] Department of Biomedical Engineering, Yale University, New Haven, CT; [2] Perlmutter Cancer Center, New York University Langone Medical Center, New York, NY; [3]Department of Medicine, New York University School of Medicine, New York, NY; [4]Department of Surgery, Yale University, New Haven, CT; [5]Department of Molecular Biophysics and Biochemistry, Yale University, New Haven, CT; [6]Department of Pathology, Yale University, New Haven, CT; [7]Department of Surgery, University of Cambridge, Cambridge, UK; [8]Department of Immunobiology, Yale University, New Haven, CT; [9]Department of Biochemistry and Molecular Pharmacology, New York University School of Medicine, New York, NY; * co-corresponding authors: Shohei.Koide@nyulangone.org and gregory.tietjen@yale.edu

Supplementary Figures

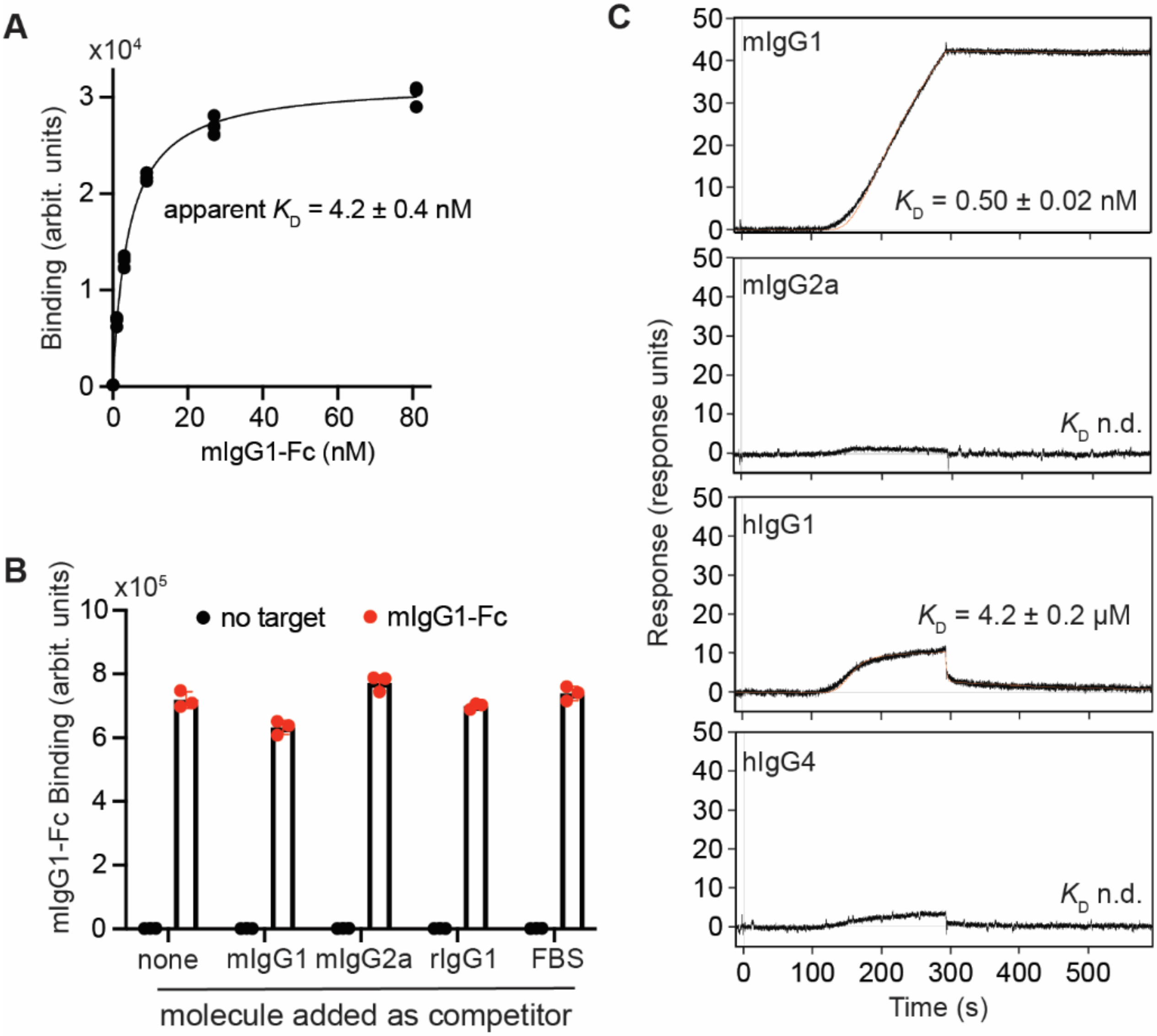

Supplementary Figure 1. Characterization of FCM101. (A) Binding titration of FCM101 to mIgG1-Fc in the yeast display format. Binding signal of biotinylated mouse IgG1-Fc was detected using streptavidin-DyLight650 using flow cytometry. The apparent K_D value shown is the mean and SD (n = 3 independent binding tests). (B) Binding competition experiment of FCM101 in the yeast display format. Yeast cells displaying FCM101 were first mixed with mIgG1-Fc (50

nM), and then mixed with the equal volume of buffer containing the indicated competitors. The final concentrations were 1 µM for IgGs and 20% for FBS. rIgG, rat IgG. After incubation for 1 hr at 37 °C, the bound biotinylated mIgG1-Fc was quantified as in (A) but on a different instrument (the values are therefore not comparable as they are). The black dots show signals without mIgG1-Fc, corresponding to complete displacement. (C) Surface plasmon resonance sensorgrams showing the association and dissociation of IgG samples to surface-immobilized FCM101 measured on a Pioneer instrument (Sartorius). The OneStep gradient injection of the analytes was used. The experimental data are shown in black and thin red lines show the best fit of the 1:1 binding model. The errors shown are from curve fitting. n.d., not determined because the signals were too weak for curve fitting.

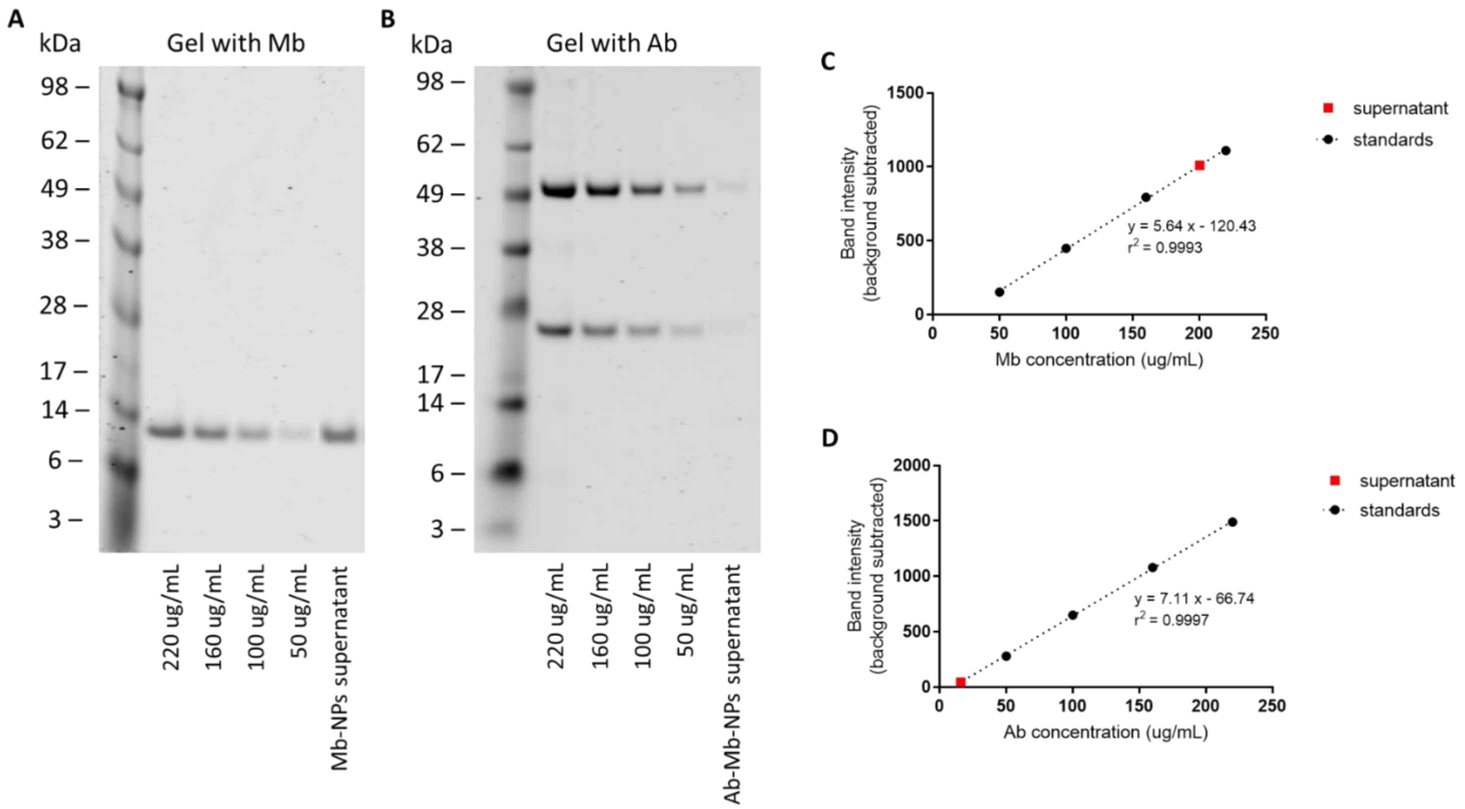

Supplementary Figure 2. **Generation of standard curves to quantify antibody and monobody concentrations in the supernatant.** (A, B) Images of an SDS-PAGE gel containing the concentration standards of Mb (A) or Ab (B) and a sample of the supernatant post Mb conjugation (A) or Ab association to the Mb-NPs (B) demonstrating the size and charge separation of Mb and Ab in the gels. The uncropped gels can be found in the source data file. (C, D) Graphs of a representative standard curve for the Mb gel (C) or Ab gel (D) with the equation and the correlation coefficient of the fitted linear curve used to determine the concentration in the samples of unknown concentration (supernatant). Mb, monobody; Ab, antibody; NP, nanoparticle.

A

	maleimide-NP	Mb-NP	Ab-Mb-NPs	COOH-NP	Ab-NP
Hydrodynamic diameter	164 ± 27 nm [n=9]	162 ± 13 nm [n=9]	168 ± 23 nm [n=37]	173 ± 12 nm [n=6]	165 ± 20 nm [n=4]
PdI	0.16 ± 0.04 [n=9]	0.15 ± 0.04 [n=9]	0.15 ± 0.04 [n=37]	0.14 ± 0.01 [n=6]	0.15 ± 0.01 [n=4]
Zeta potential	-5 ± 3 mV [n=9]	-14 ± 5 mV [n=9]	-12 ± 3 mV [n=37]	-32 ± 4 mV [n=6]	-17 ± 6 mV [n=4]
Conjugation efficiency	-	38 ± 9 % [n=18]	84 ± 6 % [n=18]	-	47 ± 9 % [n=2]
Mass of Mb / mg of NP	-	38 ± 9 ug [n=18]	-	-	-
Mass of Ab / mg of NP	-	-	47 ± 3 ug [n=18]	-	23 ± 5 ug [n=2]

B

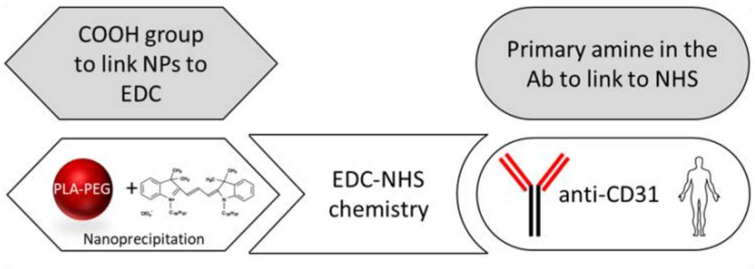

C

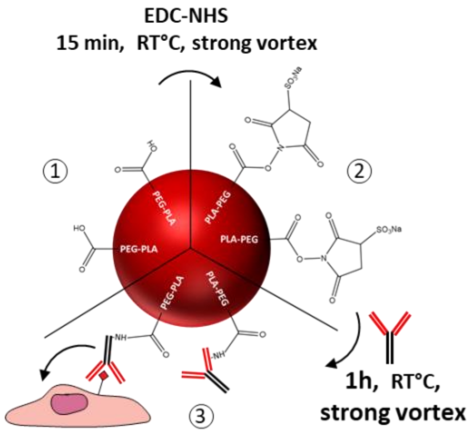

Supplementary Figure 3. Antibody coupled to the surface of nanoparticles through primary amines chemistry (EDC-NHS) compared with Mb-adaptor method. (A) Values of diameter, polydispersity index (PdI) and zeta potential measured by dynamic light scattering (DLS) at each step of the formulation. Mass antibody (Ab) per mg of nanoparticle (NP) and the conjugation efficiency were calculated from the concentration in the conjugation supernatant determined by SDS page electrophoresis. n is the number of measurements performed on independent batches to give an average and a standard deviation. (B, C) Schematics illustrating the conjugation of an antibody to the surface of a nanoparticle through primary amines chemistry (EDC-NHS). RT, room temperature; h, hour; EDC, 1-ethyl-3-(3-dimethylaminopropyl) carbodiimide hydrochloride; NHS, N-hydroxysuccinimide.

Parameters used for the calculation	
NPs diameter	100 nm
NPs density	1.25 g/cm^3
Mb molecular weight	13.4 kDa
Ab molecular weight	150 kDa
PLA-PEG-maleimide molecular weight	21 kDa

Results calculated for the NPs	
Surface area of 1 NP	31416 nm^2/NP
Number of NPs per mg	1.53 10^{12} NP/mg
Number of maleimide per NP	9.38 10^3 mal/NP

Results calculated for the Mb and Ab	
Number of Mb per NP	1120 Mb/NP
Number of Mb per surface area	3.56 10^{-2} Mb/nm^2
Number of Ab per NP	123 Ab/NP
Number of Ab per surface area	3.93 10^{-3} Ab/nm^2
Maleimide to Mb ratio	8.4 mal/Mb
Mb to Ab ratio	9 Mb/Ab

Supplementary Table 4. Table of parameters used in nanoparticle surface area and concentration calculations. Ab, antibody; Mb, monobody; NP, nanoparticle; PLA-PEG, Poly (lactic acid)-poly (ethylene glycol).

A

	Mb-NP		Ab-Mb-NPs	
	Small batches	Large batches	Small batches	Large batches
Hydrodynamic diameter	162 ± 14 nm [n=7]	153 ± 18 nm [n=11]	163 ± 32 nm [n=16]	150 ± 10 nm [n=12]
PdI	0.16 ± 0.03 [n=7]	0.17 ± 0.04 [n=11]	0.15 ± 0.05 [n=16]	0.16 ± 0.04 [n=12]
Zeta potential	-11 ± 3 mV [n=10]	-7 ± 2 mV [n=7]	-10.7 ± 2.3 mV [n=16]	-6.5 ± 0.9 mV [n=12]
Conjugation efficiency	41 ± 8 % [n=10]	35 ± 4 % [n=7]	83 ± 5 % [n=18]	82 ± 1 % [n=4]
Mass of Mb / mg of NP	41 ± 8 ug [n=10]	35 ± 4 ug [n=7]	-	-
Mass of Ab / mg of NP	-	-	47 ± 3 ug [n=18]	46 ± 1 ug [n=4]

B

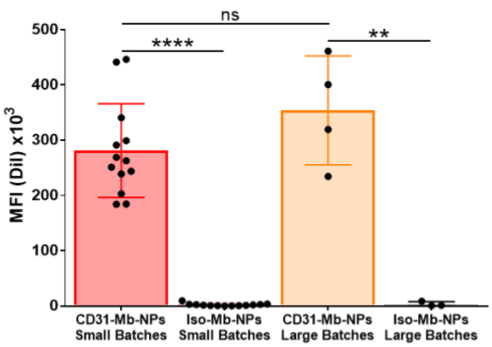

Supplementary Figure 5. Ab-Mb-NPs batches of different size can be easily prepared without characteristic alteration. (A) Values of diameter, polydispersity index (PdI) and zeta potential measured by dynamic light scattering (DLS) at each step of the formulation. Mass antibody (Ab) per mg of nanoparticle (NP) and the conjugation efficiency were calculated from the concentration in the conjugation supernatant determined by SDS page electrophoresis. n is the number of measurements performed on independent batches to give an average and a standard deviation (B) summary data of the mean fluorescence intensity (MFI) measured by flow cytometry of human umbilical vein endothelial cells (HUVECs) after incubation. Each data point represents the averaged mean fluorescent intensity (MFI) from fluorescent NPs in 3 wells with 10,000 HUVECs captured/well (n = at least 3 independent experiments). Statistical significance is shown between groups with **** where p<0.001, ** where p< 0.01, and ns where no significant difference was found using multiple two-tailed T-tests with a Bonferroni correction. Data are presented as mean values +/- SD. Mb, monobody.

	PACE-NPs	Mb-PACE-NPs	Ab-Mb-PACE-NPs
Hydrodynamic diameter	301 ± 65 nm [n=3]	308 ± 77 nm [n=2]	327 ± 44 nm [n=8]
PdI	0.16 ± 0.08 [n=3]	0.19 ± 0.11 [n=2]	0.18 ± 0.04 [n=8]
Zeta potential	16 ± 6 mV [n=3]	-3± 1 mV [n=2]	-16 ± 13 mV [n=8]
Conjugation efficiency	-	28 ± 5 % [n=2]	24 ± 11 % [n=8]
Mass of Mb / mg of NP	-	14 ± 3 ug [n=2]	14 ± 3 ug [n=2]
Mass of Ab / mg of NPs	-	-	13 ± 6 ug [n=8]

Supplementary Table 6. PACE-NPs characterization at each step of the conjugation with monobody and antibody. Values of diameter, polydispersity index (PdI) and zeta potential measured by dynamic light scattering (DLS) at each step of the formulation. Mass Ab per mg of NP and the conjugation efficiency were calculated from the concentration in the conjugation supernatant determined by SDS page electrophoresis. n is the number of measurements performed on independent batches to give an average and a standard deviation. Ab, antibody; Mb, monobody; NP, nanoparticle; PACE, Poly(amine-co-ester).

	Single	Single	Pair 1		Pair 2	
	K2	K3	K5	K6	K7	K8
Donor age (years)	82	62	65		49	
Donor sex	M	M	M		F	
Donor type (DBD/DCD)	DBD	DCD	DBD		DBD	
Left/Right kidney	Left	Left	Left	Right	Left	Right
Reason for decline	Chronic bilateral renal lobulation with cortical thinning	Biopsy revealed a moderate to severe arterial sclerosis with hyaline	High terminal creatinine		Possible malignancy	
Medical history	-	0-5 years history htn compliant with Tx, heavy cigarette and EtOH use	-		multiple medical comorbidities (asthma, CHF, DM, HTN, OSA, obesity)	
Cause of death	stroke	Head trauma	-		Anoxia	
Cold ischemia time	6h	18h30	18h	18h	8h14	8h14
KDPI	-	84	-		76	
Organ delivered on	Hypothermic machine perfusion	Hypothermic machine perfusion	Hypothermic machine perfusion	Hypothermic machine perfusion	Static cold storage	Static cold storage
Blinded and randomized (Yes/No)	No	No	Yes	Yes	Yes	Yes
Type NPs of NPs injected	CD31-Mb-NPs	Iso-Mb-NPs	CD31-Mb-NPs	Iso-Mb-NPs	Iso-Mb-NPs	CD31-Mb-NPs

	Pair		Single
	K11	K12	K13
Donor age (years)	70		68
Donor sex	M		M
Donor type (DBD/DCD)	DCD/DBD		DBD
Left/Right kidney	Right	Left	Right
Reason for decline	Abdominal aorta heavily calcified		Anatomical/CIT
Medical history	Barretts oesophagus, liver disease, high alcohol use		-
Cause of death	Cardiac arrest		Intracranial hemorrhage
Cold ischemia time	14h		13h
KDPI	-		-
Organ delivered on	Static cold storage		Static cold storage
Blinded and randomized (Yes/No)	Yes	Yes	Yes
Type NPs of NPs injected	CD31-Mb-NPs	CD31-NPs (EDC-NHS coupled)	CD31-Mb-NPs (red) & CD31-NPs (green)

Supplementary Table 7. Donor demographics of the transplant-declined human kidney enrolled in the study. DBD, donation after brain death; DCD, donation after circulatory death; HTN, hypertension; Tx, treatment; EtOH use, alcohol use; CHF, congestive heart failure; DM, Diabetes Mellitus; OSA, obstructive sleep apnea; CIT, cold ischemia time; h = hour; iso, isotype; Mb, monobody; NP, nanoparticle; EDC, 1-ethyl-3-(3-dimethylaminopropyl) carbodiimide hydrochloride; NHS, N-hydroxysuccinimide.

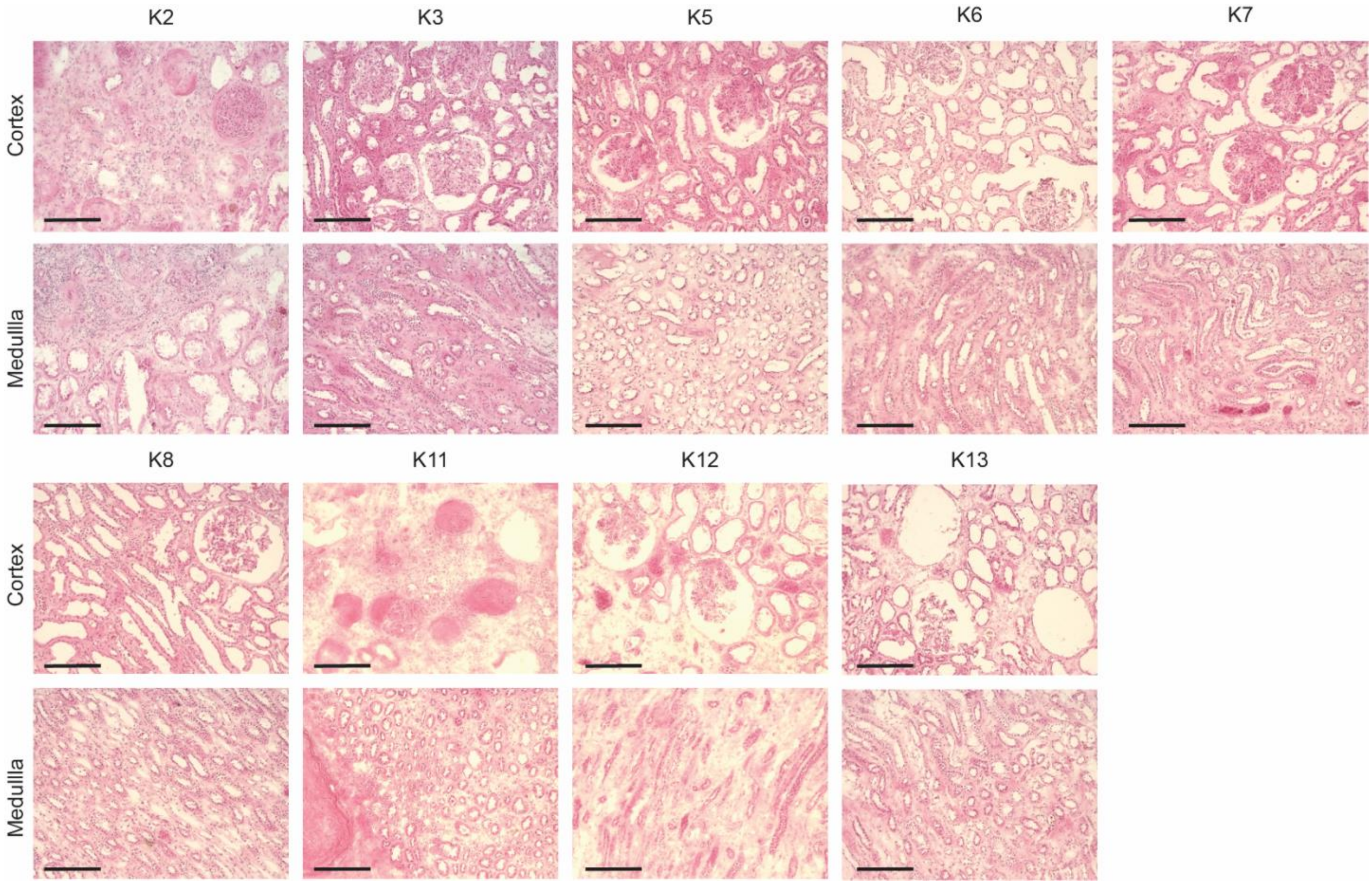

Kidney ID	Glomerulosclerosis	Interstitial Fibrosis/ Tubular Atrophy	Arteriosclerosis	Hyalinosis	Tubular injury
K2	>10%	11-20% of tissue	mild intimal fibrosis + mild intimal thickening	moderate	severe
K3	<5%	1-10% of tissue	mild intimal sclerosis	0	severe
K5	<5%	1-10% of tissue	mild intimal sclerosis	0	severe
K6	<5%	1-10% of tissue	mild intimal sclerosis	0	severe
K7	<5%	1-10% of tissue	none	0	severe
K8	<5%	1-10% of tissue	none	0	severe
K11	5-10%	1-10% of tissue	mild intimal sclerosis	0	severe
K12	5-10%	1-10% of tissue	mild intimal sclerosis	0	severe
K13	<5%	1-10% of tissue	none	0	severe

Supplementary Figure 8. Histological samples from the transplant-declined human kidneys enrolled in the study and pathology report. Frozen tissue sections were stained with hematoxylin and eosin. Scale bar is 200μm. The table includes blinded sample scoring based on 3 slices of tissue from each of three biopsies per kidney. 20 images were taken for each tissue slice.

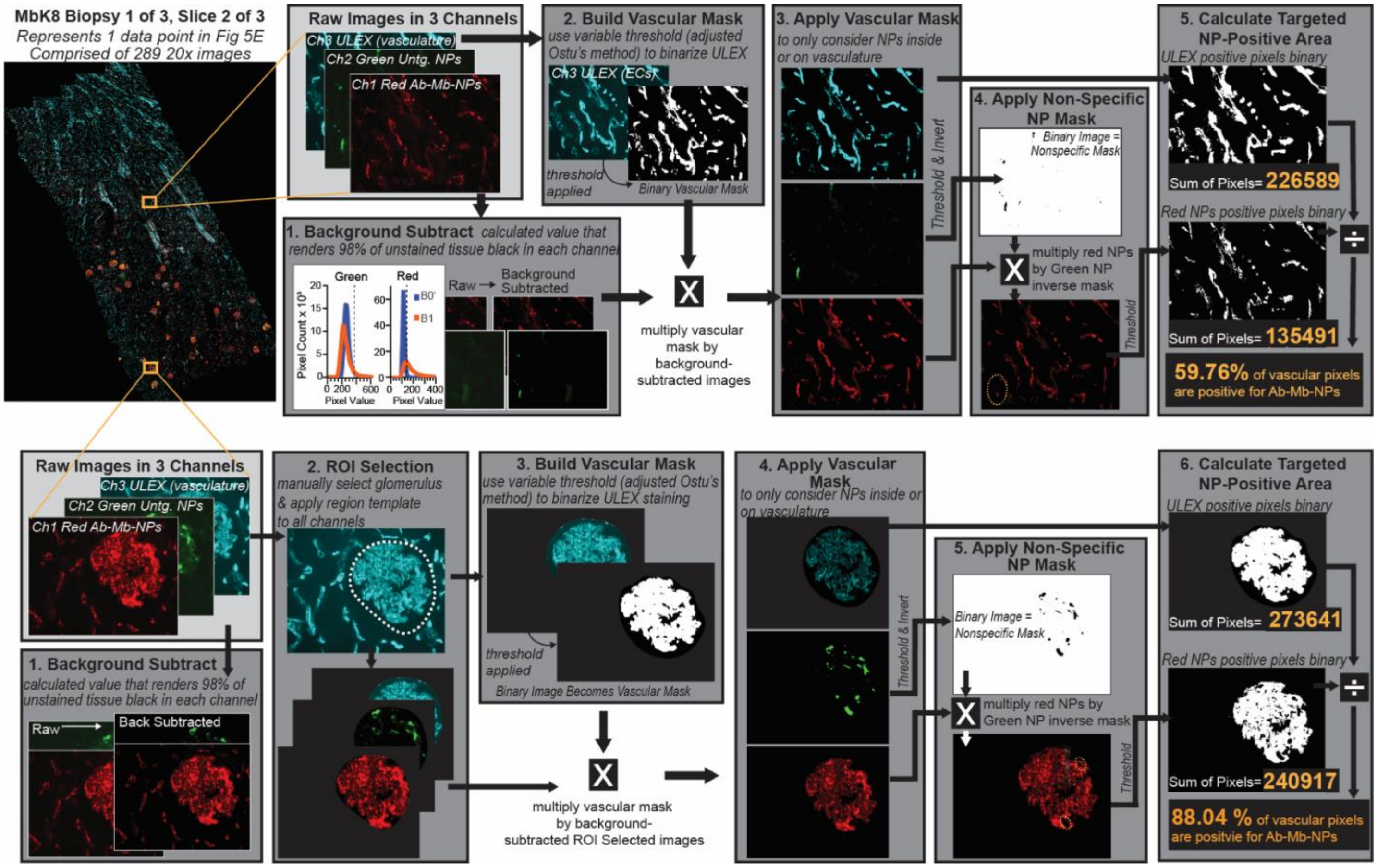

Supplementary Figure 9. Schematics of the image processing for the human kidney samples. NP, nanoparticle; Ab, antibody; Mb, monobody; untg, untargeted.

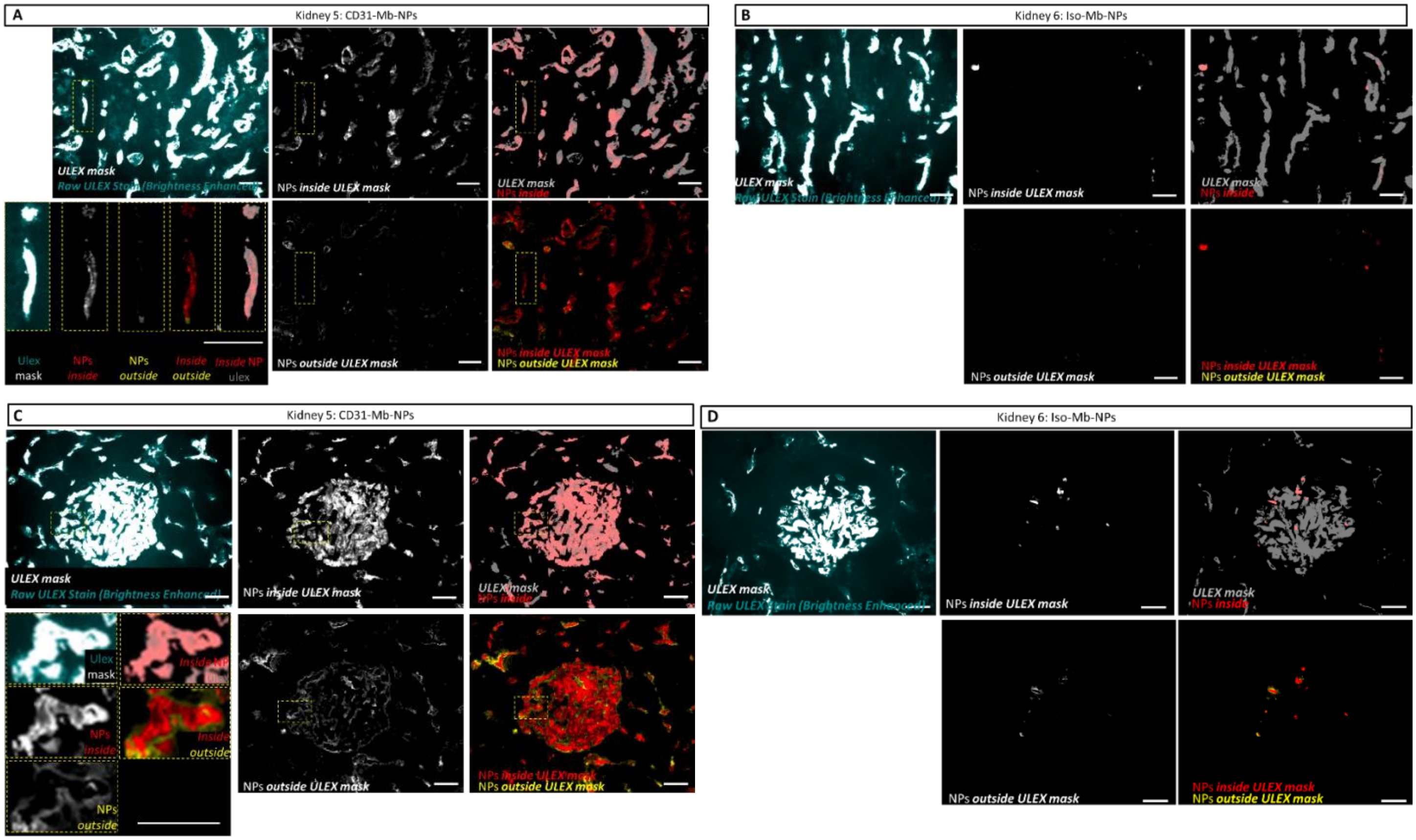

Supplementary Figure 10. Colocalization of ULEX staining and nanoparticle signal. (A-D) Representative high magnification images of microvasculature and glomeruli from perfused kidneys from Kidney 5 (A and C) and Kidney 6 (B and D). Images are representative of 300 images for the microvessels (A and B) and 30 images for the glomeruli (C and D) repeated three times per kidney biopsy. Scale bar is 50um. Within each panel: Overlay of uncorrected, brightness enhanced ULEX staining (cyan) with the ULEX mask used for future analysis (white). In this overlay, some regions of weak/dim ULEX staining are not captured in the mask, which is built using the best fit threshold for the image on the whole and captures the majority of the vasculature. This minor exclusion could be due to incomplete ULEX penetration of the 10µm tissue slices, resulting in dimmer signal on deeper vascular structures. Images of targeted NP signal that occurs within the ULEX staining mask, and an overlay of nanoparticle (NP) signal inside the ULEX mask (red) with the ULEX mask itself (gray), demonstrates the pixel colocalization of these two signals. Images of targeted NP signal that occur outside the ULEX staining mask, and an overlay of NP signal that occurred inside (red) and outside (yellow) the ULEX mask. In this overlay, it is apparent that the majority of the NPs are detected within positive ULEX staining. Additionally, those that were detected outside the ULEX mask are proximal to ULEX positive structures. The exclusion of these NPs could be due to incomplete ULEX mask coverage the depth of tissue where the NPs are located. This observation is clear in higher magnification views of a particular vascular structures, which are selected from the images and outlined in yellow dashes. In these, NPs detected outside the ULEX mask occur near the edge of the mask, and these regions correspond to dimly stained tissue that was not captured in the mask, likely uncaptured vasculature.

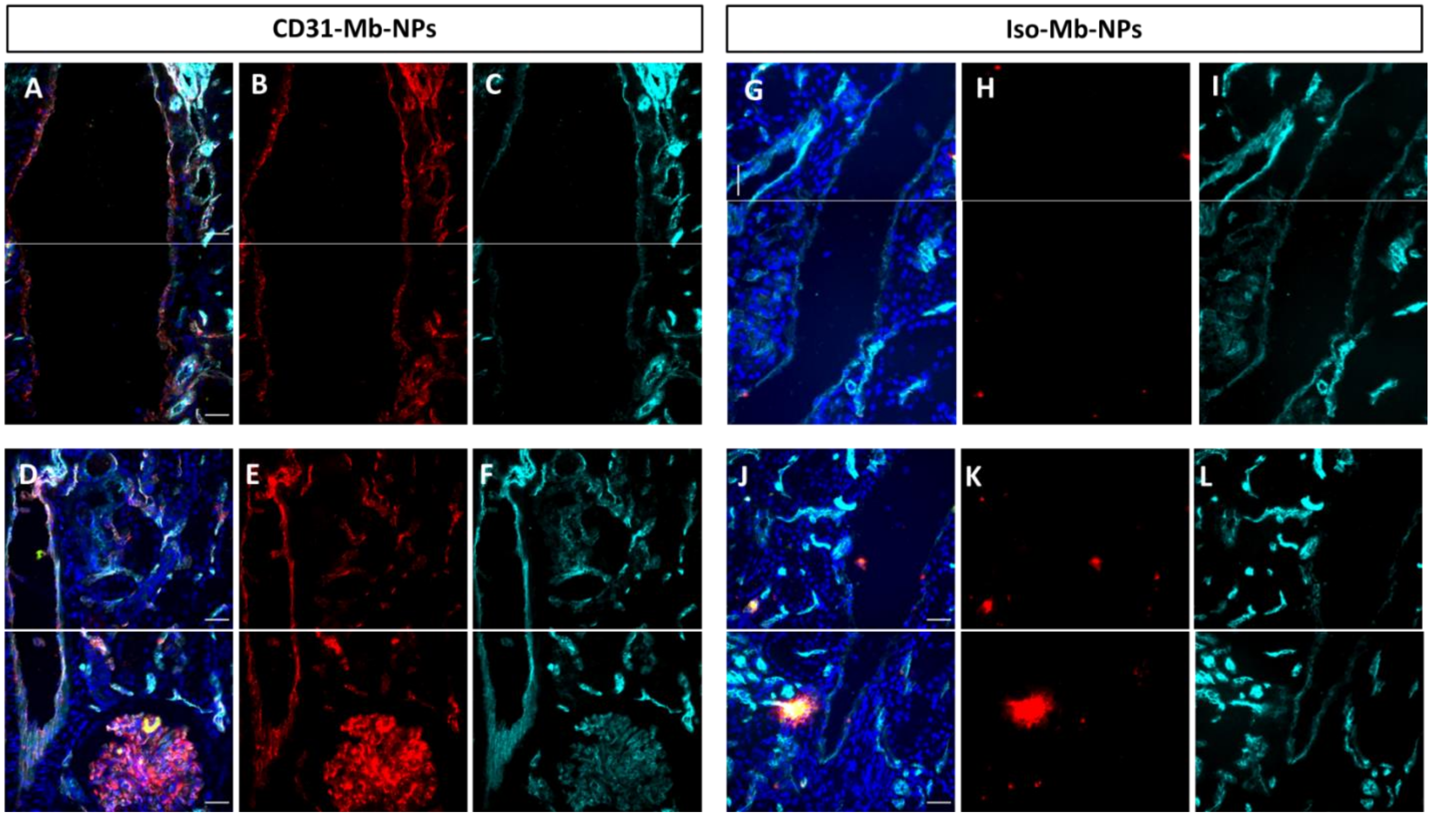

Supplementary Figure 11. CD31-Mb-NP accumulation on large vessels in human kidney. (A-F) Representative images of a large diameter vessel from Kidney 8 Biopsy 1, each tiled together from two 20x images, showing a merged view (A, D) with ULEX vascular stain in cyan, Ab-Mb-NPs in red, untargeted NPs in green, and DAPI nuclear stain in dark blue, and individual color images of Ab-Mb-NPs (B, E) and ULEX vascular stain (C, F). (G-L) Representative images of a large diameter vessel from Kidney 7 Biopsy 1, each tiled together from two 20x images, with the same color organization. Images are representative of 300 images repeated three times per kidney biopsy. Scale bars are all 50um. NP, nanoparticle; Ab, antibody; Mb, monobody.

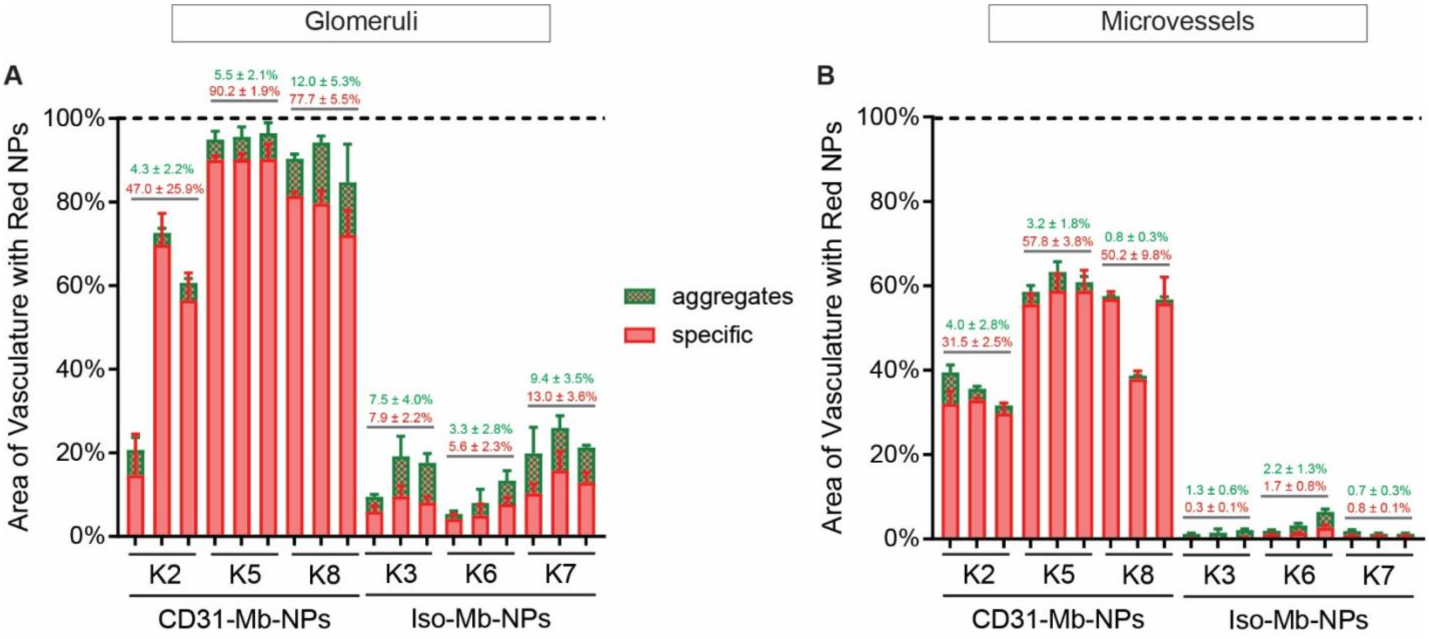

Supplementary Figure 12. Vascular coverage by specific nanoparticle binding and nonspecific accumulation in red blood cell clots. (A,B) Summary quantification of the specific, red nanoparticle (NP) coverage on microvascular structures (A) and glomeruli (B), together with the specific, red NPs that colocalized with untargeted, green NPs (n = 3 tissue slices). The presence of red blood cell clots (detected by green NP physical entrapment) resulted in the removal of colocalized targeted, red NPs from analysis in Figure 5, since there is no way to determine if those NPs bound specifically to endothelial cells with attached antibodies (Abs), or if they were physically trapped. A consideration of all the targeted NPs found however demonstrates that near 100% coverage was reached in some glomeruli samples. As analyzed in Figure 5, three biopsies were taken from each of 6 kidneys after the 4 hour ex vivo perfusion. Three slices of 10μm thickness, separated by 100μm, were sampled from each biopsy. Each data point represents the area of vasculature determined to have NP presence by fluorescence signal in a slice (approximately 45 mm^2 area, or 300, 20x images) (A) or approximately 24 glomeruli (B), divided by the total area of vasculature. Data are presented as mean values +/- SD. Iso, isotype; Mb, monobody.

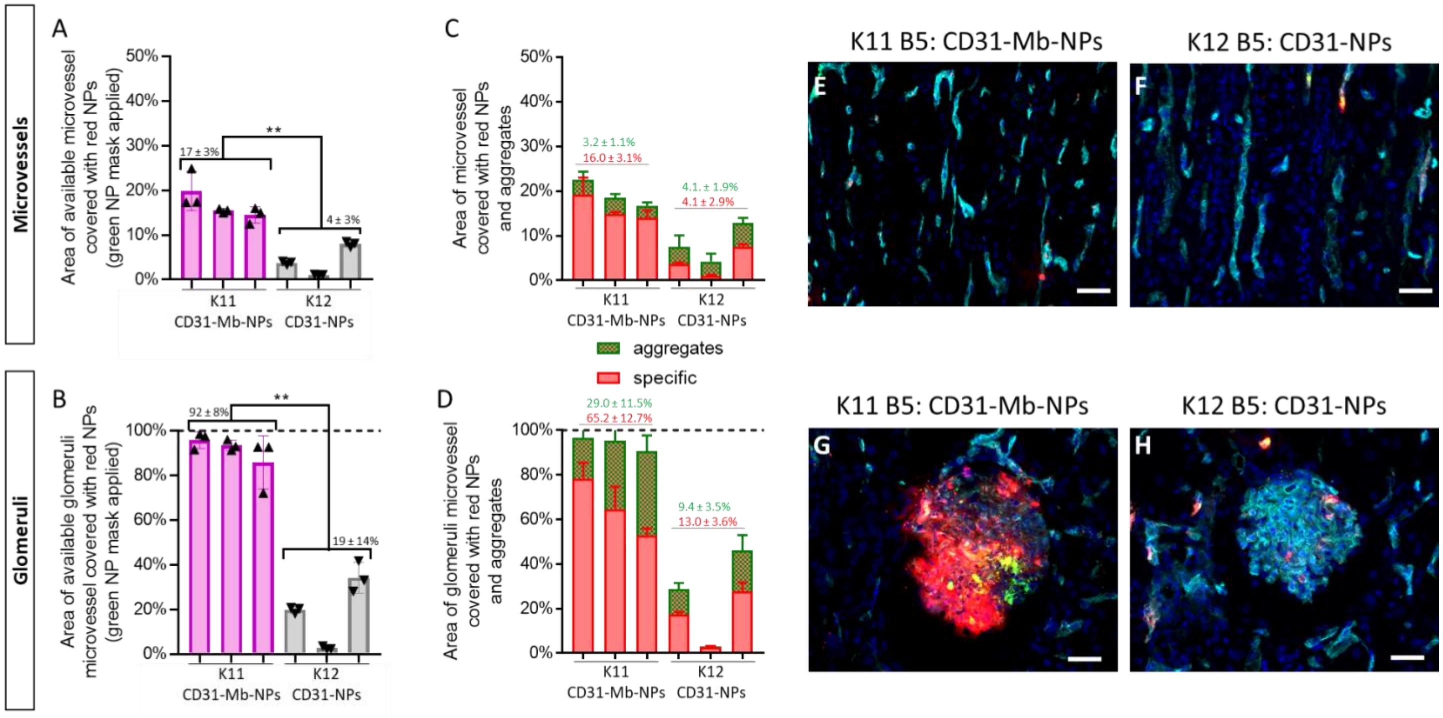

Supplementary Figure 13. The monobody adapter improves binding potency in human renal endothelium during ex vivo organ perfusion. (A, B) Summary quantification of the red nanoparticle (NP) coverage on available microvascular structures (A) and glomeruli (B). CD31-Mb-NPs and CD31-NPs with the same fluorescent dye were injected in a pair of kidneys (MbK11 and MbK12). MbK11 received red-labeled CD31-Mb-NPs and MbK12 received red-labeled CD31-NPs, both received green-labeled untargeted NPs that were used to identify blockages in the vasculature due to the red blood cells aggregates. They were compared with the same methodology used to compare CD31-Mb-NPs and Iso-Mb-NPs in Figure 5. Three biopsies were taken from each kidney after the 4 hour ex vivo perfusion. Three slices of 10µm thickness, separated by 100µm, were sampled from each biopsy. (C, D) Summary quantification of the specific, red NP coverage on total microvascular structures (C) and glomeruli (D), together with the red NPs that colocalized with untargeted green NPs identified as NPs stuck in red blood cell aggregates. A consideration of all the red NPs (specific + aggregates) found demonstrates that near 100% coverage was reached in glomeruli samples. Each data point represents the area of vasculature determined to have NP presence by fluorescence signal in a slice (approximately 45 mm^2 area, or 300, 20x images) (A, C) or approximately 30 glomeruli (B, D), divided by the total area of vasculature (n = 3 tissue slices). Statistical significance between treatments is denoted with **, $p < 0.01$, by an unpaired two-tailed T-test. Data are presented as mean values +/- SD. (E-H) Representative high magnification images of microvasculature and glomeruli display NPs in red, ULEX vascular stain in cyan, cell nuclei in dark blue, and untargeted NPs in green. Images are representative of 300 images for the microvessels (E and F) and 30 images for the glomeruli (G and H) repeated three times per biopsy. Scale bar is 50 µm. Kidneys perfused with CD31-Mb-NPs have much higher NP coverage (E, G) compared with kidneys perfused with CD31-NPs (F, H). Source data are provided as a Source Data file. Iso, isotype; Mb, monobody.

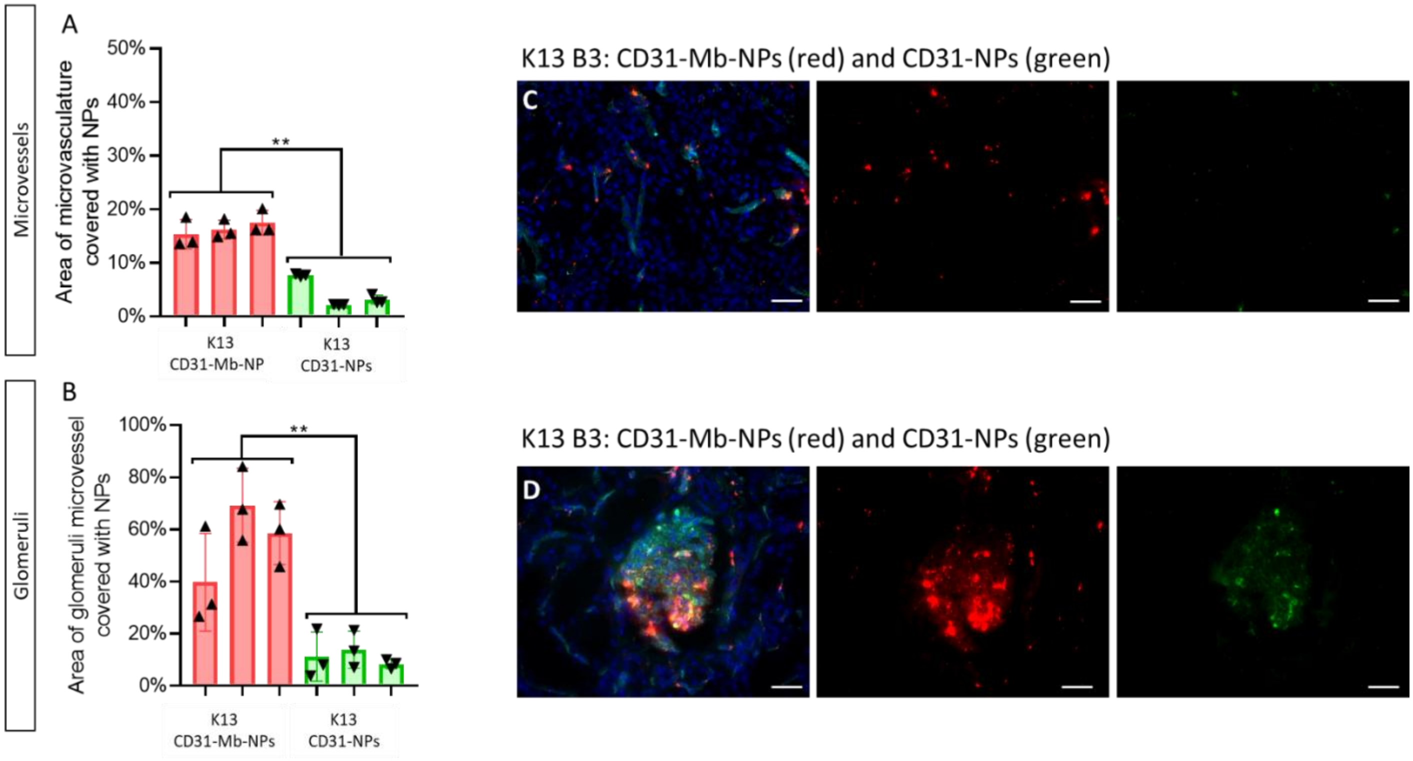

Supplementary Figure 14. The monobody adapter results in superior binding potency in human renal endothelium during ex vivo organ perfusion when compared with other targeted nanoparticles. (A, B) Summary quantification of the nanoparticle (NP) coverage on microvascular structures (A) and glomeruli (B) (n = 3 tissue slices). Red labeled CD31-Mb-NPs and green labeled CD31-NPs were injected together in a single kidney (MbK13). Three biopsies were taken from the kidney after the 4 hour ex vivo perfusion. Three slices of 10µm thickness, separated by 100µm, were sampled from each biopsy. Statistical significance between treatments is denoted with **, $p < 0.01$, by an unpaired two-tailed T-test. Data are presented as mean values +/- SD. (C, D) Representative high magnification images of microvasculature and glomeruli display CD31-Mb-NPs in red, CD31-NPs in green, vascular cells in cyan, and cell nuclei in dark blue. The first image is a merged display, with individual red and green channel images to the right. Images are representative of 300 images for the microvessels (C) and 30 images for the glomeruli (D) repeated three times per kidney biopsy. Scale bar is 50 µm. Source data are provided as a Source Data file. Mb, monobody.

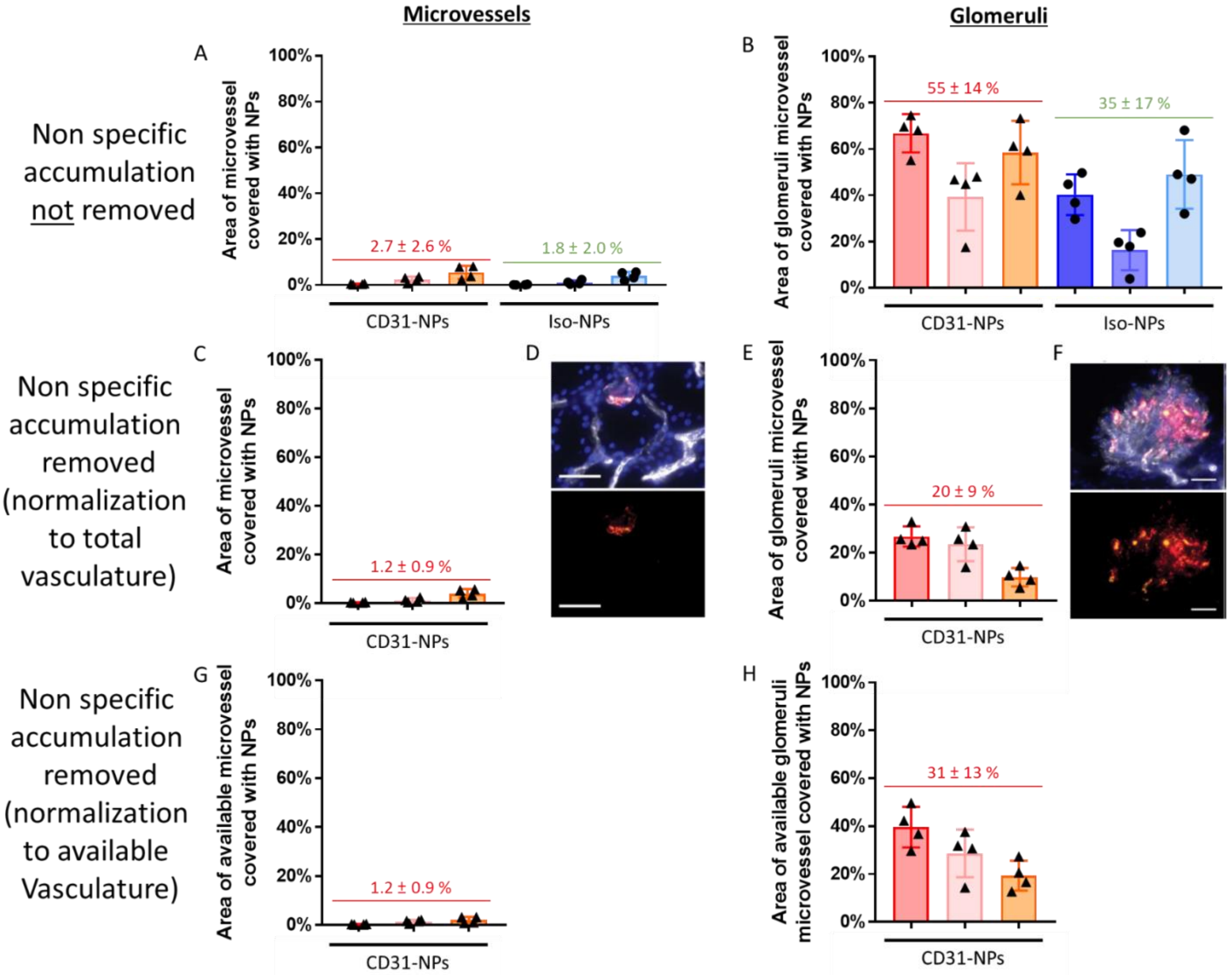

Supplementary Figure 15. Reanalysis of the images from the previous study using Ab-NPs. (A, B) Summary quantification of the CD31-NPs (red) and Iso-NPs (green) coverage on microvascular structures (A) and glomeruli (B) without subtraction of non-specific accumulation. (C & E) Summary quantification of the CD31-NPs (red) coverage on microvascular structures (C) and glomeruli (D) after subtraction of area of non-specific accumulation (green Iso-NPs mask applied). .(G & H) Summary quantification of the CD31-NPs (red) coverage on available microvascular structures (G) and glomeruli (H) after subtraction of area of non-specific accumulation (green Iso-NPs mask applied). One biopsy was taken from each of the 3 kidneys after the 4 hour ex vivo perfusion. 4 serial slices of 20µm thickness were sampled from each biopsy. Each data point represents the area of vasculature determined to have NP presence by fluorescence signal in a slice (10 images taken at 20x) (A, C, G) or approximately 10 glomeruli (B, E, H), divided by the total area of vasculature (A,B,C,E) or by the available area of vasculature (i.e. the total area of vasculature minus the area of vasculature occupied by unspecific NPs) (G, H) (n = 4 tissue slices). Data are presented as mean values +/- SD. (D & F) Representative images of microvasculature (D) and glomeruli (F) with high specificity reproduced from Tietjen et al – 2017. Images are representative of 10 images for the microvessels (D) and 10 images for the glomeruli (F) repeated four times per kidney biopsy. Scale bar is 50 µm. NP, nanoparticle; Ab, antibody; iso, isotype.

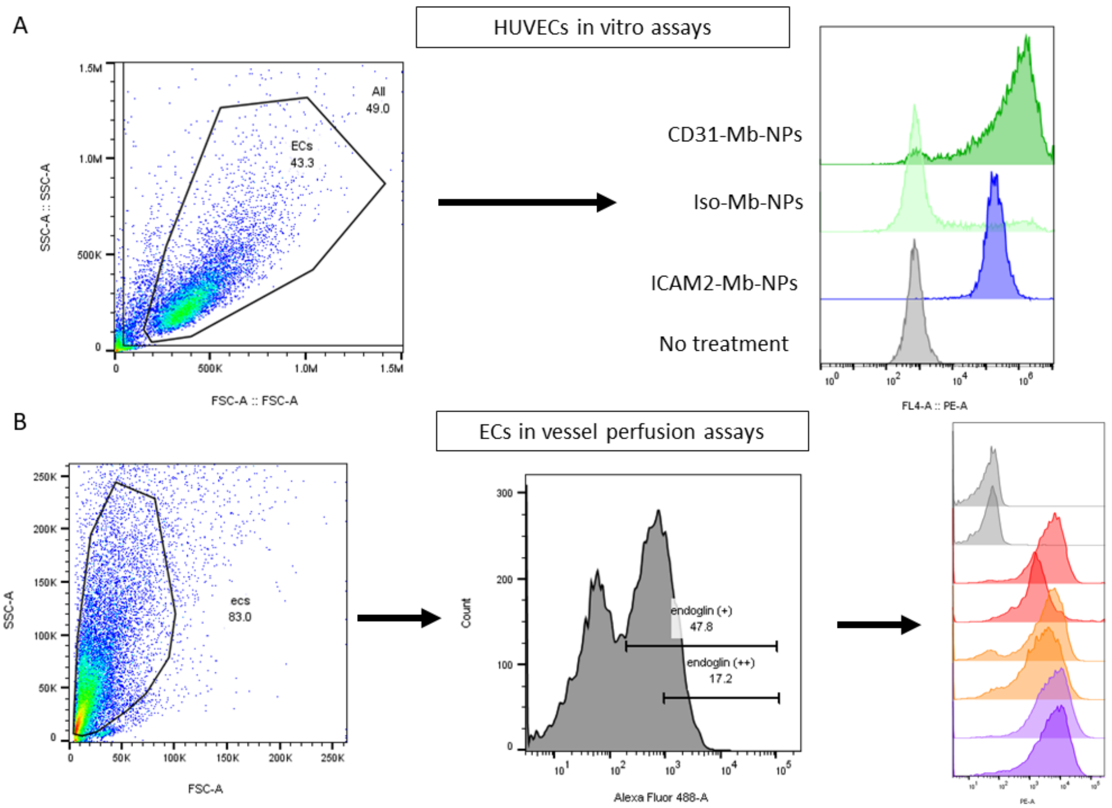

Supplementary Figure 16. Gating strategies for human umbilical vein endothelial cells and isolated endothelial cells from vessel perfusions. Example analysis of human umbilical vein endothelial cells (A) and isolated endothelial cells (ECs) (B) performed in FlowJo for flow cytometry data. In both analyses, the EC population was first defined using a forward and side scatter plot to collect cells of the appropriate size. *In vitro* cells could then be directly measured for PE (fluorescent nanoparticle) signal (A). *Ex vivo* cells were further gated on positive signal for FITC-tagged anti-endoglin antibody to identify ECs from the mixed population. FSC, forward scatter; SSC, side scatter.
