# Supplementary material for: Monobody adapter for functional antibody display on nanoparticles for adaptable targeted delivery applications
Source: Nat Commun. 2022 Oct 11;13:5998. doi: 10.1038/s41467-022-33490-8 (PMC9553936; doi:10.1038/s41467-022-33490-8)
Supplement: Supplementary file 3 — Reporting Summary [file 41467_2022_33490_MOESM3_ESM.pdf]

nature portfolio

Corresponding author(s): Gregory T. Tietjen & Shohei Koide

Last updated by author(s): 2022/09/20

Reporting Summary

Nature Portfolio wishes to improve the reproducibility of the work that we publish. This form provides structure for consistency and transparency in reporting. For further information on Nature Portfolio policies, see our Editorial Policies and the Editorial Policy Checklist.

Statistics

For all statistical analyses, confirm that the following items are present in the figure legend, table legend, main text, or Methods section.

n/a	Confirmed	
☐	☒	The exact sample size (*n*) for each experimental group/condition, given as a discrete number and unit of measurement
☐	☒	A statement on whether measurements were taken from distinct samples or whether the same sample was measured repeatedly
☐	☒	The statistical test(s) used AND whether they are one- or two-sided<br>*Only common tests should be described solely by name; describe more complex techniques in the Methods section.*
☒	☐	A description of all covariates tested
☐	☒	A description of any assumptions or corrections, such as tests of normality and adjustment for multiple comparisons
☐	☒	A full description of the statistical parameters including central tendency (e.g. means) or other basic estimates (e.g. regression coefficient) AND variation (e.g. standard deviation) or associated estimates of uncertainty (e.g. confidence intervals)
☐	☒	For null hypothesis testing, the test statistic (e.g. *F*, *t*, *r*) with confidence intervals, effect sizes, degrees of freedom and *P* value noted<br>*Give P values as exact values whenever suitable.*
☒	☐	For Bayesian analysis, information on the choice of priors and Markov chain Monte Carlo settings
☒	☐	For hierarchical and complex designs, identification of the appropriate level for tests and full reporting of outcomes
☒	☐	Estimates of effect sizes (e.g. Cohen's *d*, Pearson's *r*), indicating how they were calculated

Our web collection on statistics for biologists contains articles on many of the points above.

Software and code

Policy information about availability of computer code

Data collection	Flow cytometry data was collected using CytExpert Acquisition and Analysis Software Version 2.4 (Beckman Coulter), BD FACSDiva™ (BD Biosciences), or ForeCyt v8.0 (iQue). Fluorescence images were collected using EVOS FL Auto 2 Software (ThermoFisher Scientific), ZEN pro (Zeiss), or Micro-Manager Open Source Microscopy Software. CryoEM images were obtained using SerialEM (open source, MIT license, Version 3.9 beta). Nanoparticle physical characteristics were measured using Zetasizer Advance (Malvern Panalytical). SPR data was collected using Pioneer software v4.3.1.
Data analysis	Flow cytometry data was analyzed using FlowJo v10. Fluorescence images were analyzed using ImageJ 1.5.2 (NIH) and a custom MATLAB program. CryoEM images were processed for visualization using RELION (3.1beta-fosscuda-2018b-2020-0617). SPR data was analyzed using QDAT software v4.3.1. SDS gel images were analyzed using Image Studio Lite (Li-COR Biosciences). Mathematical and statistical analysis was performed in Excel (Microsoft) and Graphpad Prism v8.

For manuscripts utilizing custom algorithms or software that are central to the research but not yet described in published literature, software must be made available to editors and reviewers. We strongly encourage code deposition in a community repository (e.g. GitHub). See the Nature Portfolio guidelines for submitting code & software for further information.

Data

Policy information about availability of data

All manuscripts must include a data availability statement. This statement should provide the following information, where applicable:

- Accession codes, unique identifiers, or web links for publicly available datasets
- A description of any restrictions on data availability
- For clinical datasets or third party data, please ensure that the statement adheres to our policy

The data generated in this study are provided in the Supplementary Information and Source Data files. Raw images are available upon request due to file size limitations.

Field-specific reporting

Please select the one below that is the best fit for your research. If you are not sure, read the appropriate sections before making your selection.

☒ Life sciences ☐ Behavioural & social sciences ☐ Ecological, evolutionary & environmental sciences

For a reference copy of the document with all sections, see nature.com/documents/nr-reporting-summary-flat.pdf

Life sciences study design

All studies must disclose on these points even when the disclosure is negative.

Sample size	Cell culture experiments routinely are performed in triplicate wells per experiment (with at least 10,000 cells per well) and each experiment was repeated at least 3 times as determined by previous experience to obtain consistent results. To determine 10,000 cells per well is sufficient, experiments were conducted measuring the signal from a fluorescent CD31 Ab 2,000 - 100,000 cells per well which is expected to be uniform and consistent. 10,000 cells per well produced consistent values with minimal deviation among the population. More experiments were included as time and supplies allowed. Human vessel experiments included at least 3 vessels per experimental group, as determined by Lysyy and Bracaglia, Bioengineering & Translational Medicine 2020, to provide consistent data. Human kidney experiments were conducted on 6 organs, which was determined by a power analysis using a two-sample T test and previously published data describing nanoparticle signal in perfused human kidneys (alpha = 0.05, expected relative NP binding signal difference of 10.5, standard deviation of 3.5, and power of 0.95).
Data exclusions	No data was excluded from the analysis.
Replication	Each experiment was performed at least 3 times at separate occasions. The reproducibility was confirmed and is shown in the data representations.
Randomization	All allocation of cell culture and human vessel experiments was random. The first two human kidneys obtained were assigned either targeted nanoparticles or control nanoparticles (non-randomized) to provide additional insight into experimental design before proceeding with additional samples. Each subsequent kidney was assigned to either the targeted or control nanoparticle group in random, double-blinded process.
Blinding	Cell culture and human vessel experiments were partially blinded. The experimenter knew the identity of the NPs added to each specimen, which was allowed for logistical purposes. However, the state of the cells, vessels, and NPs to be tested were unknown to the experimenter until the time of the analysis. These variables are what might lead to biased results. For instance, even though the identity of the NPs is known, certain ones cannot be added to a “better” vessel or cell population, since this information is not knowable. After the first two kidneys (which were not randomized as described above), nanoparticle groups were assigned in a double-blinded process to ensure non-biased group assignment.

Reporting for specific materials, systems and methods

We require information from authors about some types of materials, experimental systems and methods used in many studies. Here, indicate whether each material, system or method listed is relevant to your study. If you are not sure if a list item applies to your research, read the appropriate section before selecting a response.

Materials & experimental systems

n/a	Involved in the study
☐	☒ Antibodies
☐	☒ Eukaryotic cell lines
☒	☐ Palaeontology and archaeology
☒	☐ Animals and other organisms
☐	☒ Human research participants
☒	☐ Clinical data
☒	☐ Dual use research of concern

Methods

n/a	Involved in the study
☒	☐ ChIP-seq
☐	☒ Flow cytometry
☒	☐ MRI-based neuroimaging

Antibodies

Antibodies used

Purified anti-human-CD31 monoclonal antibody (PECAM-1, IgG1 mouse, clone WM59, Cat. 96246, Lot# B300520), purified mouse IgG1 K isotype control antibody (clone MOPC-21, Cat. 95923, Lot# B300504) were purchased from Biolegend. Purified and FITC labeled anti-porcine-CD31 monoclonal antibody (PECAM-1, IgG1 mouse, clone LCI-4, Purified Cat. MA5-28345, FITC Cat. 11-0319-42 & Lot# 2031862), purified anti-human-CD102 monoclonal antibody (ICAM-2, IgG1 mouse, clone BT-1, Cat. MA1-34500, Lot# SK247611113), Alexa Fluor 488 labeled anti-human-CD105 monoclonal antibody (endoglin, IgG1 mouse, clone SN6, Cat. MHCD10520, Lot# 2100140) FITC labeled anti-human-CD31 monoclonal antibody (PECAM-1, IgG1 mouse, clone WM59, Cat. 11-0319-42, Lot# 2171939 & 2031862), Alexa Fluor 647 labeled rabbit anti-Mouse IgG (H+L) cross-adsorbed secondary antibody (Cat. A21239, Lot# 1954174) were acquired from Invitrogen. BUV395 labeled anti-human-CD105 monoclonal antibody (endoglin, IgG1 mouse, clone 266, Cat. 563803, Lot# 0316750) was obtained from BD Bioscience.

Validation

Biolegend reports for CD31 wm59 & IgG1 MOPC-21 that each lot of this antibody is quality control tested by immunofluorescent staining with flow cytometric analysis. Invitrogen reports that FITC labeled anti-human-CD31 (Cat. 11-0319-42) has been pre-titrated and tested by flow cytometric analysis of normal human peripheral blood cells. All other antibodies from Invitrogen list flow cytometry as a tested application and include referenced publications using the antibody. Invitrogen also states that they used cell treatment (endoglin, MHCD10520) or immunofluorescence (ICAM2, MA1-34500) to verify that the antibody binds to the correct antigen. BD reports that anti-human-CD105 (Cat. 563803) is routinely tested with flow cytometry. We independently verified that the anti-porcine-CD31 antibody bound to porcine aortic endothelial cells before using it to target NPs (data included). Experiments which use antibodies for tissue identification or for cell gating are tested against isotype controls in all cases. In all experiments when antibodies were used to target NPs, each antibody reagent was also verified for consistent cell binding using a secondary antibody for detection.

Eukaryotic cell lines

Policy information about cell lines

Cell line source(s)

Human Umbilical Vein Endothelial Cells (HUVECs) were obtained from the Yale Vascular Biology and Transplantation tissue culture core laboratory, where they were isolated from fresh umbilical veins by treatment with collagenase. Porcine Aortic Endothelial Cells (PAECs) were purchased from Cell Applications, Inc.

Authentication

PAECs were authenticated by Cell Applications, Inc by testing positive for DiI-Ac-LDL uptake as well as being able to attach, spread, and proliferate in Growth Medium (bioassay). HUVECs were not authenticated but were derived from fresh human umbilical chord on site. Additionally, the nanoparticle targeting experiments typically used CD31, a canonical endothelial cell marker.

Mycoplasma contamination

PAECs tested negative for mycoplasma contamination (testing done by Cell Applications, Inc). HUVECs were not tested for mycoplasma contamination.

Commonly misidentified lines
(See ICLAC register)

No commonly misidentified cell lines were used.

Human research participants

Policy information about studies involving human research participants

Population characteristics

Donor kidneys organs were obtained as available from consenting organ donors. The details of the 6 donors included in this study are described in the manuscript. Briefly, donors are aged 49-82 yrs old with various co-morbidities contributing to the decision to decline each organ for transplant and make available for research. In this study, 5 of the 6 donors were men. 4 donors were DCD (donation after cardiac death), 1 donor was DBD (donation after brain death), and 1 donor was DBD/DCD. Each kidney made available for research that was able to be perfused ex vivo in a consistent and stable manner was used in this study.

Recruitment

Potential organ donors are identified by organ procurement organization representatives. All organs are first made available to transplant centers, and upon universal decline, can be recovered for research. From this available pool, a donor demographic exclusion criteria was defined to eliminate organs that were physically unable or challenging to be perfused ex vivo. This exclusion criteria has age and co-morbidity dependencies (as noted in the manuscript) but if an organ passed exclusion criteria, no additional efforts were made to distribute age, sex, race, etc and the included population based on the

organ availability.

Ethics oversight

All necessary ethical approvals were obtained in advance of receiving any organ. Informed consent was obtained from the donor's family according to New England Donor Services policy and standing policies within the UK's National Health Service. In the U.S., we have consulted with our local IRB at Yale University who has informed us that organs recovered from deceased donors do not represent human subjects research according to the definition for Human Subjects in the HHS Policy for Protection of Human Research Subjects 45 CFR 46.102: "Human subject means a living individual about whom an investigator (whether professional or student) conducting research obtains." HIPAA is still invoked and we have a HIPAA Privacy Form on file.

Note that full information on the approval of the study protocol must also be provided in the manuscript.

Flow Cytometry

Plots

Confirm that:

☒ The axis labels state the marker and fluorochrome used (e.g. CD4-FITC).

☒ The axis scales are clearly visible. Include numbers along axes only for bottom left plot of group (a 'group' is an analysis of identical markers).

☒ All plots are contour plots with outliers or pseudocolor plots.

☒ A numerical value for number of cells or percentage (with statistics) is provided.

Methodology

Sample preparation

Human umbilical vein endothelial cells were obtained from human umbilical veins and cultured up to passage 5. Porcine aortic endothelial cells were purchased from Cell Applications and cultured until passage 5. For flow cytometry, cells were cultured in 96 well plates at a final density of 40,000 cells/well. After the experimental procedure, cells are lifted from the plate using 40uL/well TrypLE™ Express Enzyme (Gibco Cat No. 12605036). The trypsin was neutralized with 100uL cell media and the lifted cells were stored on ice until flow cytometric analysis (less than 2 hours). Cells that were obtained directly from umbilical ateries (after perfusion) were collected by first filling the vessel with a 0.1% collagenase IV solution, and incubating the vessel for 10 minutes at 37C. Cells are then flushed from the vessel using 2 mL of PBS + 1% BSA, and washed using additional PBS+ 1% BSA. Cells are then stained and stored on ice until analysis (less than 2 hours).

Instrument

Flow cytometry was conducted on either the CytoFLEX Flow Cytometer (Beckman Coulter) or the LSRII (BD Biosciences).

Software

Data was collected using CytExpert Acquisition and Analysis Software Version 2.4 (Beckman Coulter) or BD FACSDiva™ (BD Biosciences). Data was analyzed using FlowJo v10.

Cell population abundance

For cell culture experiments, each well was sampled to obtain 10,000 cells within an endothelial-cell-sized-gate. This population was verified to be >99% CD31+ cells (an endothelial cell marker) when taken from consistent cell culture protocols. In tissue analysis, all cells obtained from a vessel segment were sampled, and sorted by CD105+ staining to obtain endothelial cells. This resulted in ~30,000 endothelial cells per segment.

Gating strategy

Endothelial cells were first gated by FSC and SSC selection, with a polygon gate drawn at approximately 10k and 30k in FSC and 10k to 30k in SSC, to eliminate debris. In cell culture experiments, no additional gates were drawn, and NP signal could be evaluated in the whole endothelial cell population. In tissue experiments, whole cells fitting into the above described gates were evaluated for CD105+ staining. Positive staining was determined using a control cell population, stained with an isotype Ab of the same fluorophore, and drawing a gate which excludes all nonspecific or background fluorescence. From this population of endothelial cells, NP signal could be evaluated.

☒ Tick this box to confirm that a figure exemplifying the gating strategy is provided in the Supplementary Information.
